# Effects of different frequencies of ankle pump exercise on lower limb hemodynamics: a systematic review and meta-analysis

**DOI:** 10.3389/fphys.2026.1880108

**Published:** 2026-07-30

**Authors:** Zixin He, Chunhua Liu, Zegen Ye, Zhi Ye, Yongfei Zheng, Zunjing Zhang

**Affiliations:** Lishui Hospital of Traditional Chinese Medicine Affiliated to Zhejiang University of Chinese Medicine, Lishui, Zhejiang, China

**Keywords:** ankle pump exercise, deep vein thrombosis, exercise intervention, frequency, venous hemodynamics

## Abstract

**Background:**

Ankle pump exercise (APE) is commonly used to improve lower-limb circulation, but the hemodynamic effects of different frequencies remain unclear. This study evaluated frequency-specific hemodynamic changes after APE to inform practical exercise protocols.

**Methods:**

As of March 2026, six databases—PubMed, Embase, the Cochrane Library, CNKI, Wanfang, and VIP—were systematically searched according to PRISMA guidelines. Eight studies involving 546 participants, including healthy individuals and patients with postoperative immobilization, fractures, hemiplegia, or restricted mobility, were included, of which only one was an RCT. The effects of APE at different frequencies on mean femoral venous blood flow velocity were analyzed using a paired change-score approach. Analyses were conducted at the specific-frequency level to reduce unit-of-analysis error from repeated frequency comparisons and shared baseline measurements. Sensitivity analyses were performed, and methodological quality was assessed using the NHLBI quality assessment tools according to study design.

**Results:**

Studies were grouped according to APE frequency, and frequency-specific within-arm analyses showed that mean femoral venous blood flow velocity generally increased from baseline across all evaluated frequencies. Smaller increases were observed at lower frequencies, particularly at 3 cycles/min, whereas more pronounced within-arm increases were generally observed at 10 cycles/min or higher, including at 10 cycles/min (MD = 15.16 cm/s, 95% CI: 11.87 to 18.46). However, this observation was based mainly on indirect comparisons of changes from baseline across studies rather than direct head-to-head comparisons between different frequencies. No consistent pattern indicated that progressively higher frequencies necessarily resulted in additional benefits. Sensitivity analyses using fixed-effect models and leave-one-out procedures showed that the direction and statistical significance of the findings were generally stable.

**Conclusions:**

APE may improve lower-limb venous hemodynamics by increasing mean femoral venous blood flow velocity. Frequencies of 10 cycles/min or higher tended to show larger within-arm increases than lower-frequency protocols, but this observation was based mainly on indirect cross-study comparisons rather than direct head-to-head comparisons and should be interpreted cautiously. Given the predominance of non-randomized or quasi-experimental studies and the very low certainty of evidence, the optimal exercise frequency remains uncertain, and further well-designed studies are needed to confirm these findings.

## 1 Introduction

Deep vein thrombosis (DVT) is a major component of venous thromboembolism and a common vascular complication in hospitalized patients (Replantation of severed limbs. Analysis of 40 cases, 1975). Epidemiological evidence indicates that DVT remains a substantial global health burden, with an annual incidence of roughly 53–162 cases per 100, 000 people in the general population (Wolf et al., 2024). DVT not only increases the risks of disability, recurrence, and death, but also contributes to longer hospital stays, greater healthcare utilization, and higher economic costs (Shahi et al., 2017). In patients with prolonged bed rest, postoperative immobilization, critical illness, or restricted mobility, reduced lower-limb venous flow and blood stasis are more common, increasing the risk of thrombosis and potentially delaying recovery and subsequent rehabilitation (Zhao et al., 2021; Ruan et al., 2023). Therefore, improving lower-limb circulation and venous return is of clear clinical importance for DVT prevention and rehabilitation.

Despite the established role of anticoagulant therapy in the prevention of DVT, its clinical application is often restricted by bleeding risk, contraindications during the perioperative period, and poor suitability for certain patient populations (Burnett et al., 2016; Moster and Bolliger, 2022). For this reason, non-drug preventive strategies remain indispensable, especially in patients with prolonged immobilization, postoperative inactivity, or impaired mobility.

Ankle pump exercise (APE) has been widely used as a convenient and accessible mechanical intervention for DVT prevention (Shi et al., 2023). Through repeated ankle movements, APE stimulates cyclical contraction of the calf musculature, thereby augmenting the muscle pump effect, facilitating venous return, and improving lower-limb hemodynamic status (Horwood, 2019). Its benefits in promoting venous circulation and alleviating stasis have led to broad application in clinical nursing and rehabilitation practice (Liu et al., 2025). However, a standardized APE regimen has not yet been defined, and the choice of exercise frequency remains particularly controversial (Li et al., 2022). While slower frequencies have traditionally been used in routine care, emerging studies suggest that faster movement patterns may be associated with more pronounced improvements in venous flow (Nakayama et al., 2016; Li et al., 2022). Given the substantial variation in tested frequencies and the inconsistency of reported findings, the optimal frequency of APE remains uncertain. Therefore, a systematic synthesis of current evidence is warranted to clarify the frequency-specific hemodynamic responses associated with APE and to provide a more reliable basis for developing practical exercise protocols.

Evidence on the effects of APE performed at different frequencies remains limited. The available studies for each specific frequency are few in number, often small in sample size, and unevenly distributed, making robust direct comparisons across individual frequencies difficult to achieve (Wang et al., 2023). In light of these evidence characteristics, the present study grouped the reported frequencies into predefined ranges for presentation and conducted analyses at the specific-frequency level to examine changes in mean femoral venous blood flow velocity after APE. This approach was intended to summarize the available evidence while avoiding overinterpretation of indirect comparisons across heterogeneous studies.

## 2 Methods

The protocol for this study was prospectively registered in PROSPERO under the identifier CRD420261360586, and the review was conducted in accordance with the PRISMA 2020 statement (Page et al., 2021).

### 2.1 Search strategy

We systematically searched PubMed, Embase, the Cochrane Library, CNKI, Wanfang, and VIP from database inception to March 1, 2026, to identify original studies on the effects of different ankle pump exercise frequencies on lower-limb hemodynamics. For PubMed and Embase, controlled vocabulary terms (MeSH and Emtree, respectively) were combined with free-text terms. The full set of search strategies is provided in **Supplementary Table 1**. In addition, the reference lists of eligible studies and relevant reviews were manually screened to identify any further potentially relevant articles.

### 2.2 Selection criteria

#### 2.2.1 Studies were eligible if they met the following criteria

(1) they were published original articles; (2) enrolled participants receiving ankle pump exercise, regardless of disease status or health condition; (3) investigated APE performed at clearly reported frequencies; (4) reported mean femoral vein blood flow velocity as an outcome, with extractable or poolable pre- and post-exercise data; (5) and used a clinical study design, including randomized controlled trials (RCTs), quasi-experimental studies, before–after studies, or other designs suitable for evaluating the effects of APE.

#### 2.2.2 Studies were excluded if they

(1) were duplicate publications; (2) were unavailable in full text; (3) did not clearly report exercise frequency; (4) lacked data on mean femoral vein blood flow velocity; (5) did not provide data suitable for extraction, conversion, or meta-analysis. Case reports, reviews, systematic reviews, conference abstracts, theses, expert opinions, animal studies, and *in vitro* studies were also excluded.

### 2.3 Data extraction

A standardized data extraction form was developed in accordance with PRISMA and tailored to the aims of this review. Two reviewers independently used this form for study screening, eligibility assessment, and data collection. Extracted information included study characteristics, participant details, ankle pump exercise protocols, and outcome data on mean femoral vein blood flow velocity before and after the intervention. When essential data required for the meta-analysis were incomplete or unavailable in the published reports, attempts were made to obtain additional information from the original authors.

After extraction, the two reviewers cross-checked all entries. Any discrepancies were resolved through discussion, and a third reviewer was consulted when necessary. Inter-reviewer agreement during the screening process was assessed using the kappa statistic.

### 2.4 Quality assessment

Given the variation in study designs, methodological quality was not judged using a single unified scale; instead, assessment tools were selected according to study type. The National Heart, Lung, and Blood Institute (NHLBI) quality assessment tool for controlled intervention studies was applied to randomized and controlled trials, while the NHLBI tool for before–and–after studies with no control group was used for single-group pre–post studies. Quality appraisal was conducted independently by two reviewers. For each domain, responses were recorded as “Yes, “ “No, “ “Cannot Determine, “ “Not Applicable, “ or “Not Reported, “ and each study was then classified overall as good, fair, or poor. Disagreements were resolved by discussion, and a third reviewer (Y.Z.) was involved when necessary.

### 2.5 Certainty of evidence

The certainty of evidence was assessed using the Grading of Recommendations Assessment, Development and Evaluation (GRADE) approach. The certainty of evidence was rated as high, moderate, low, or very low according to study limitations, inconsistency, indirectness, imprecision, and publication bias. Because the meta-analytic estimates were reported at the specific-frequency level, while the evidence was presented according to predefined frequency ranges, the GRADE assessment was summarized by predefined frequency range for the main outcome, mean femoral venous blood flow velocity.

### 2.6 Statistical methods

Meta-analyses were performed using Stata 16.0. Because mean femoral venous blood flow velocity was reported in the same unit across the included studies, effect sizes were expressed as mean differences (MDs) in change from baseline with 95% confidence intervals (CIs). For studies reporting repeated measurements before and after ankle pump exercise in the same participants, paired change scores were used rather than treating pre- and post-intervention values as independent groups. The mean change was calculated as the post-intervention mean minus the baseline mean.

When the standard deviation (SD) of the within-subject change was not reported, it was estimated from the baseline and post-intervention SDs using the following formula: SDchange = √(SDpre² + SDpost² − 2r × SDpre × SDpost), where r represents the assumed pre-post correlation coefficient. A correlation coefficient of 0.5 was used in the primary analysis, and sensitivity analyses were conducted using alternative coefficients of 0.3 and 0.7 to assess the robustness of this assumption. The standard error (SE) of the mean change was calculated as the change-score SD divided by the square root of the sample size.

The included studies were first grouped according to predefined APE frequency ranges and then analyzed at the specific-frequency level. Because several studies assessed multiple exercise frequencies in the same participants and reused shared baseline measurements, conventional overall pooled estimates across broader frequency ranges were not calculated in order to reduce unit-of-analysis error and avoid double-counting of participants. When multiple comparisons were reported in the same study, we examined whether they were derived from independent participant groups or repeated measurements in the same participants. Comparisons based on independent participant groups were treated as separate comparisons and were eligible for pooling within the same specific frequency. In contrast, comparisons derived from the same participants across different exercise frequencies were not pooled across frequency ranges. Because most included studies did not provide direct randomized or paired comparisons between different exercise frequencies, the analyses were interpreted as frequency-specific within-arm changes rather than formal comparative estimates of frequency effects. Because only one included study was an RCT and the remaining studies used non-randomized, quasi-experimental, or before-after designs, the pooled estimates were interpreted as descriptive within-arm hemodynamic changes after APE rather than causal treatment effects derived from randomized comparisons.

Considering potential clinical and methodological heterogeneity across studies, random-effects REML models were used as the primary analyses within each specific frequency when more than one eligible independent comparison was available. For frequencies represented by a single comparison, the individual mean change and 95% CI were presented descriptively. Fixed-effect inverse-variance models were additionally applied as model-based sensitivity analyses and are presented in the **Supplementary Figure**. Between-study heterogeneity was assessed within each specific-frequency analysis using Cochran’s Q test and the I² statistic, with I² values of 25%–50%, 50%–75%, and >75% indicating low, moderate, and high heterogeneity, respectively. Leave-one-out sensitivity analyses were also performed within the corresponding specific-frequency analyses, where applicable, by removing one study at a time to examine the robustness of the estimates. A two-sided P value < 0.05 was considered statistically significant. The findings were presented as forest plots.

A formal assessment of publication bias was not carried out because the number of included studies was small. According to the Cochrane Handbook, methods such as funnel plots and statistical tests for asymmetry are not recommended when fewer than 10 studies are available (Sterne et al., 2011).

## 3 Results

### 3.1 Selection of studies

**Figure 1:**
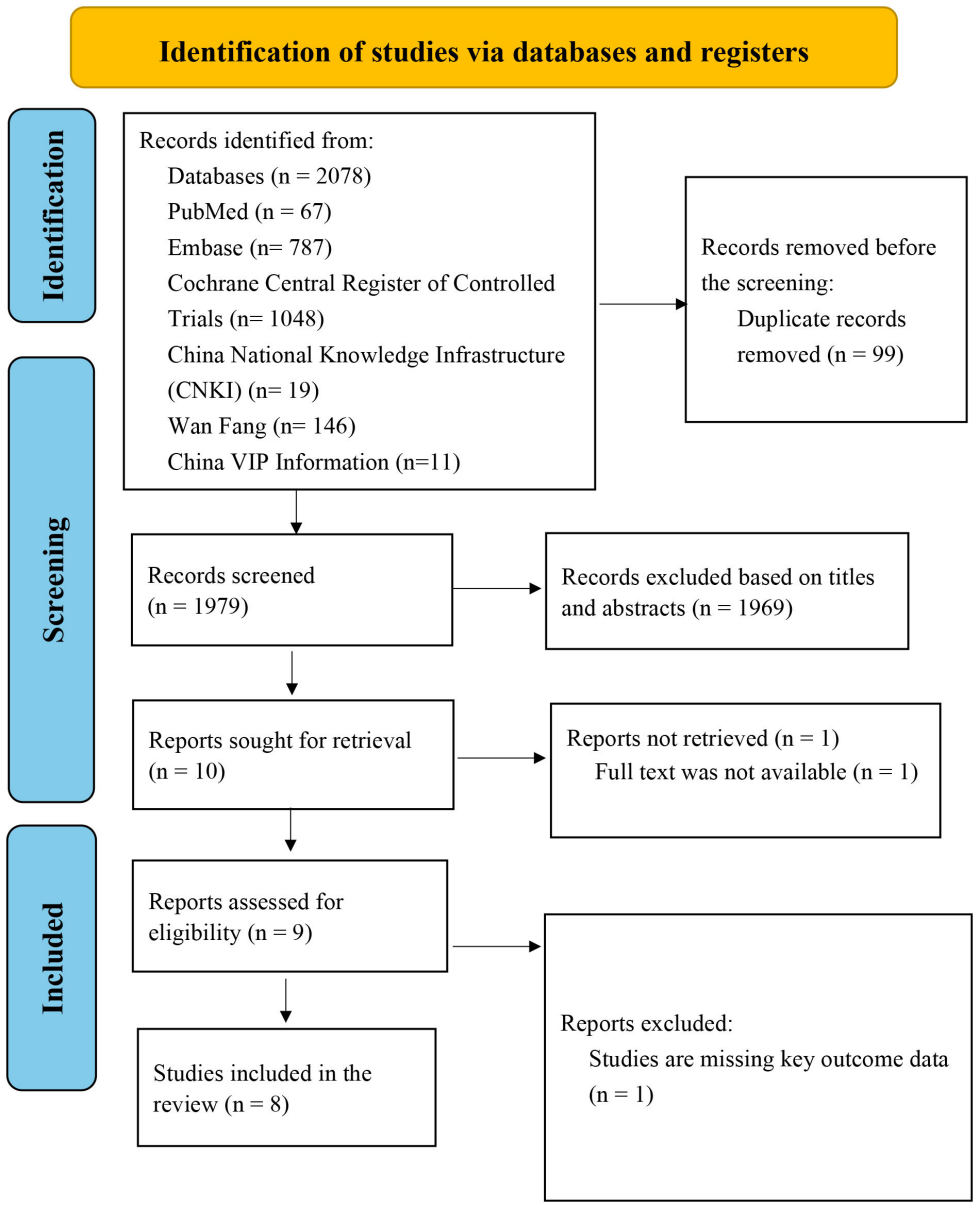
PRISMA flow diagram showing the process of study screening and selection.

A total of 2, 078 records were identified through database searching, and 1, 979 remained after removal of 99 duplicates. Following title and abstract screening, 1, 969 records were excluded, leaving 10 reports sought for retrieval. Of these, 1 report could not be retrieved because the full text was unavailable. Therefore, 9 full-text articles were assessed for eligibility. After full-text assessment, 1 article was excluded because it did not report the primary outcome. Ultimately, 8 original clinical studies were included in the meta-analysis. No additional eligible studies were identified through manual searching or reference screening. The study selection process is shown in **Figure 1**. Inter-reviewer agreement was high, with a kappa value of 0.87 (96.98% agreement) for title and abstract screening and 0.94 (99.6% agreement) for full-text assessment.

### 3.2 Study characteristics

The characteristics of the included studies are presented in **Table 1**. The 8 studies (Kagaya, 1992; Nakayama et al., 2016; Toya et al., 2016; Li et al., 2020; Zhao et al., 2020; Zhou et al., 2020; Li et al., 2022; Han et al., 2024) were published between 1992 and 2024 and were conducted in China (n = 5), Japan (n = 2), and the United States (n = 1). Only 1 study was an RCT (Li et al., 2022), whereas the remaining studies used a pre–post intervention design. Sample sizes ranged from 12 to 307, with a total of 546 unique participants, including 337 healthy individuals, 92 patients who underwent unilateral total hip arthroplasty, 57 patients with fractures, and 60 patients with hemiplegia. Regarding sex distribution, 2 studies enrolled only male participants, 1 enrolled only female participants, and the others included both sexes. The total sample size refers to unique participants across the included studies rather than the sum of all frequency-specific comparisons; the mapping of included studies, participant groups or arms, specific exercise frequencies, and frequency-specific comparisons is provided in **Supplementary Table 3**.

**Table 1:** Baseline characteristics of the included studies and characteristics of the ankle pump exercise interventions.

Study	Country	Design	Sample size	Age (year)	Female (%)	Ankle Pump Exercise frequency
(Han et al., 2024)	China	quasi-experimental design	60	55.07 ± 9.37	15 (25.00%)	3, 6, 15, or 30 cycles/min
(Zhao et al., 2020)	China	quasi-experimental design	63	56.27 ± 10.14	NA	6, 12, 30, 40, or 60 cycles/min
(Zhou et al., 2020)	China	quasi-experimental design	11	21.6 ± 3.9	0 (0.00%)	5, 10, or 15 cycles/min
(Kagaya, 1992)	USA	quasi-experimental design	6	21-22	6 (100.00%)	20, 60, or 80 cycles/min
(Li et al., 2022)	China	RCT	307	20.39 ± 1.05	265 (86.32%)	30 cycles/min
(Li et al., 2020)	China	quasi-experimental design	60	40.80 ± 13.90	24 (40.00%)	6, 10, 30, or 60 cycles/min
(Nakayama et al., 2016)	Japan	quasi-experimental design	29	61.3 ± 11.6	27 (93.10%)	40, 60, or 80 cycles/min
(Toya et al., 2016)	Japan	quasi-experimental design	10	NA	NA	15, 30 or 60 cycles/min

RCT, Randomized Controlled Trial; NA, Not available. The female percentage was calculated as the number of female participants divided by the total sample size of each study.

The included studies reported 12 specific APE frequencies, which were grouped into four predefined frequency ranges for presentation: 3–6, 10–15, 20–40, and 60–100 cycles/min. Because some studies assessed multiple frequencies and contributed more than one frequency-specific comparison, the number of comparisons exceeded the number of studies. Therefore, the total sample size refers to unique participants across the included studies rather than the sum of all frequency-specific comparisons. All included studies reported lower-limb hemodynamic outcomes. Most studies assessed venous blood flow velocity in the lower extremities, while 1 study reported the percentage of calf muscle congestion after ankle pump exercise.

### 3.3 Quality assessment and risk of bias

All included before–and–after studies without a control group were rated as fair in methodological quality. In general, these studies were consistent with the basic structure of single-group pre–post intervention research and provided some information related to sample size. However, most did not clearly describe how sample size had been determined, and the clinical definitions of inclusion and exclusion criteria were often not sufficiently explicit. These limitations reduced overall methodological rigor, leading all uncontrolled studies to be classified as fair (**Table 2**).

**Table 2:** Methodological quality assessment of pre–post studies without a control group using the NHLBI quality assessment tool.

Study	Question or objective stated	Eligible criteria pre-specified and described	Participants representative of clinical population	All eligible participants enrolled	Sample size sufficient	Intervention Clearly described/ delivered consistently	Outcomes prespecified/valid, reliable, and consistently implemented	Blinded assessors	Dropout rate ≤ 20% or accounted for in the analysis	Statistics well described	Outcomes Tested multiple times	Analysis of individual-level data	Confounding variables measured and adjusted	Exposure/risk defined/valid and reliable, and implemented consistently	Quality rating (poor/fair /good)
(Zhao et al., 2020)	Yes	Yes	Yes	Yes	Yes	Yes	Yes	Not applicable	No	Yes	No	Not applicable	Yes	No	Fair
(Zhou et al., 2020)	Yes	Yes	Yes	Yes	Yes	Yes	Yes	Not applicable	No	Yes	No	Not applicable	Yes	No	Fair
(Kagaya, 1992)	Yes	Yes	No	Not applicable	Not applicable	Yes	Yes	Not applicable	No	Yes	No	Not applicable	Yes	No	Fair
(Nakayama et al., 2016)	Yes	Yes	Yes	Yes	Yes	Yes	Yes	Not applicable	No	Yes	No	Not applicable	Yes	No	Fair
(Toya et al., 2016)	Yes	Yes	No	Not applicable	Not applicable	Yes	Yes	Not applicable	No	Yes	No	Not applicable	Yes	No	Fair

NHLBI, National Heart, Lung, and Blood Institute.

The quality assessment of controlled intervention studies is presented in **Table 3**. The RCT conducted by Li H et al. was rated as good, indicating overall sound methodological quality (Li et al., 2022). Its main limitation, however, was the relatively small sample size, which may have reduced its ability to adequately detect the effect of higher-frequency ankle pump exercise on lower-limb venous hemodynamics. The controlled intervention study by Han J et al. was rated as poor, primarily because the intervention and control conditions were based on the affected and unaffected limbs of the same individual. Since the affected limb and the contralateral healthy limb may differ substantially in baseline function and hemodynamic status, this design limits comparability and increases the risk of bias. The remaining controlled studies were judged to be of fair quality. Detailed item-level ratings are provided in **Table 3**.

**Table 3:** Methodological quality assessment of controlled intervention studies using the NHLBI quality assessment tool.

Study	Clearly stated study design	Randomization adequate	Treatment allocation concealed	Participant and providers blinded	Assessor blinded	Baseline characteristics similar	Dropout rate ≤ 20%	Differential dropout rate ≤ 15%	High adherence to treatment	Other interventions	Outcomes valid and reliable	Sample size provided 80% power	Predefined outcome measures	Intention to treat	Confounding variables measured and adjusted	Quality rating (poor /fair/good)
(Han et al., 2024)	Yes	No	Not applicable	Not applicable	Yes	Yes	No	No	Yes	No	Yes	Yes	Yes	Yes	No	Fair
(Li et al., 2022)	Yes	Yes	Yes	Yes	Yes	Yes	No	No	Yes	No	Yes	Yes	Yes	Yes	No	Good
(Li et al., 2020)	Yes	No	NR	Not applicable	NR	Yes	No	No	Yes	No	Yes	Yes	Yes	Yes	No	Fair

NHLBI, National Heart, Lung, and Blood Institute.

### 3.4 Comparison of mean femoral vein blood flow velocity before and after intervention

#### 3.4.1 APE frequency: 3–6 cycles/min

**Figure 2:**
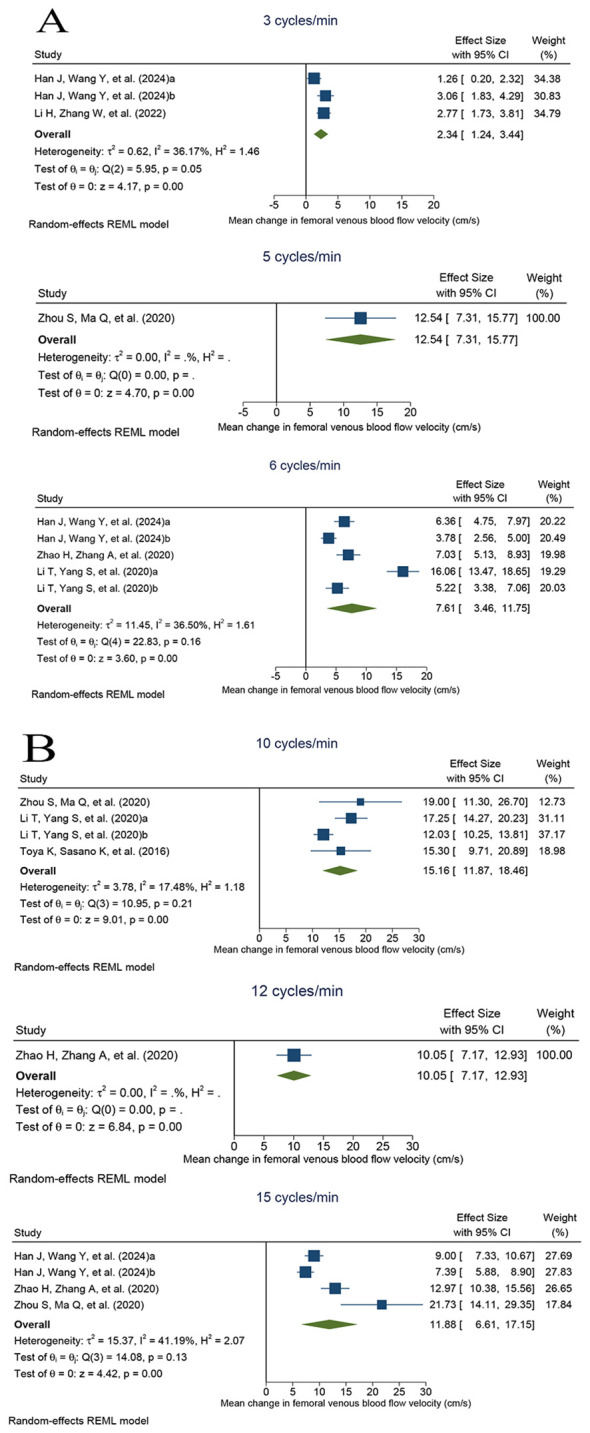
Frequency-specific changes in mean femoral venous blood flow velocity after ankle pump exercise at 3–6 and 10–15 cycles/min. **(A)** Frequency-specific changes at 3, 5, and 6 cycles/min; **(B)** frequency-specific changes at 10, 12, and 15 cycles/min. Effect estimates were calculated as paired mean changes from baseline and should be interpreted as within-frequency changes rather than formal comparative estimates between frequencies. No conventional overall pooled estimate was calculated across the broader frequency ranges to avoid unit-of-analysis error.

In the 3–6 cycles/min frequency range, analyses were conducted at the specific-frequency level for 3, 5, and 6 cycles/min using paired change-score methods and random-effects REML models where applicable. At 3 cycles/min, the pooled mean change in femoral venous blood flow velocity was 2.34 cm/s (95% CI: 1.24 to 3.44; I² = 36.17%). At 5 cycles/min, only one comparison was available, showing a mean change of 12.54 cm/s (95% CI: 7.31 to 17.77). At 6 cycles/min, the pooled mean change was 7.61 cm/s (95% CI: 3.46 to 11.75; I² = 36.50%). No conventional overall pooled estimate was calculated across the broader 3–6 cycles/min range to avoid unit-of-analysis error related to repeated frequency comparisons and shared baseline measurements (**Figure 2A**).

#### 3.4.2 APE frequency: 10–15 cycles/min

In the 10–15 cycles/min frequency range, analyses were conducted at the specific-frequency level for 10, 12, and 15 cycles/min using paired change-score methods and random-effects REML models where applicable. At 10 cycles/min, the pooled mean change in femoral venous blood flow velocity was 15.16 cm/s (95% CI: 11.87 to 18.46; I² = 17.48%). At 12 cycles/min, only one comparison was available, showing a mean change of 10.05 cm/s (95% CI: 7.17 to 12.93). At 15 cycles/min, the pooled mean change was 11.88 cm/s (95% CI: 6.61 to 17.15; I² = 41.19%). No conventional overall pooled estimate was calculated across the broader 10–15 cycles/min range to avoid unit-of-analysis error related to repeated frequency comparisons and shared baseline measurements (**Figure 2B**).

#### 3.4.3 APE frequency: 20–40 cycles/min

**Figure 3:**
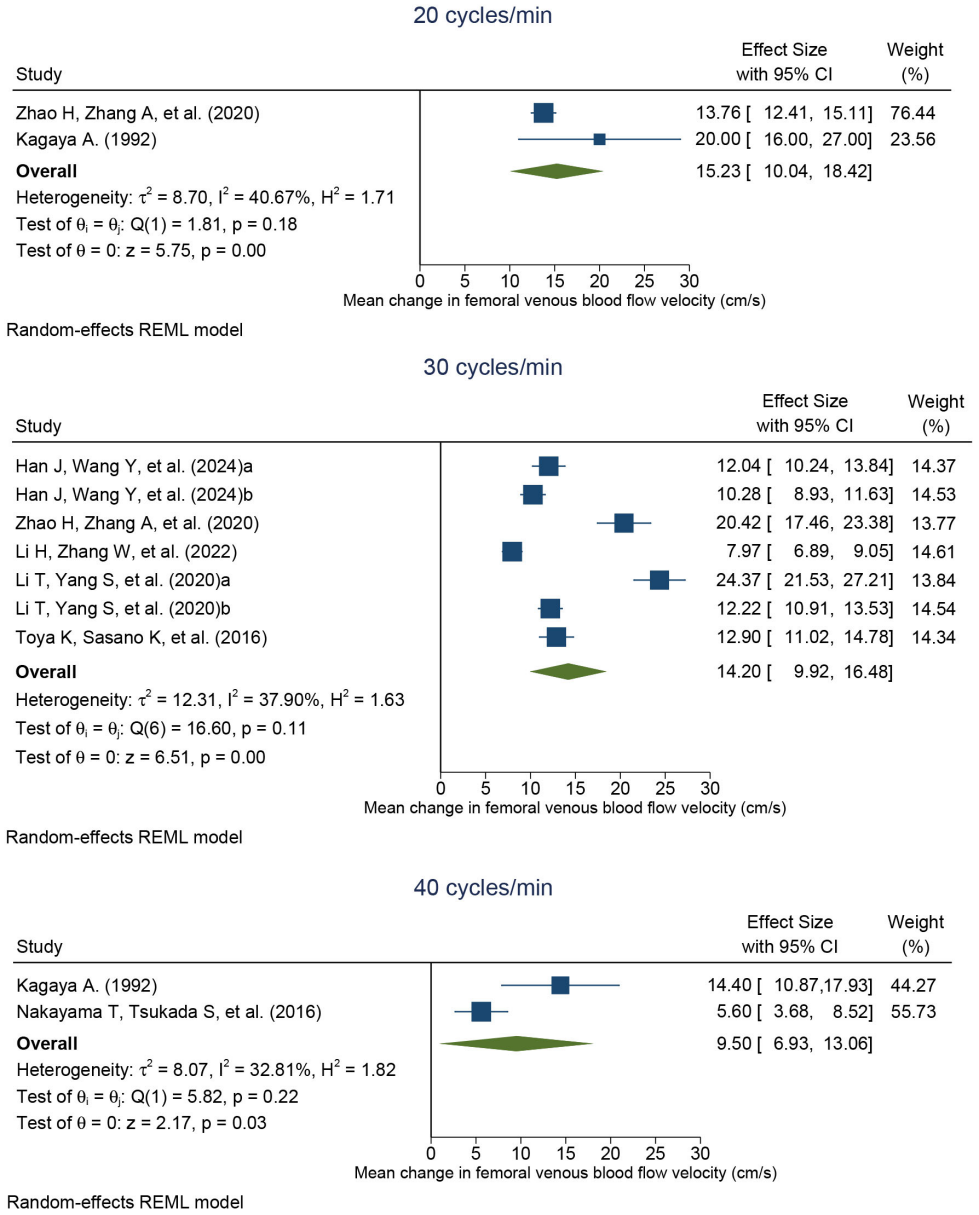
Frequency-specific changes in mean femoral venous blood flow velocity after ankle pump exercise at 20–40 cycles/min. Effect estimates were calculated as paired mean changes from baseline. Frequency-specific estimates are shown for 20, 30, and 40 cycles/min. No conventional overall pooled estimate was calculated across the broader 20–40 cycles/min range to avoid unit-of-analysis error.

In the 20–40 cycles/min frequency range, analyses were conducted at the specific-frequency level for 20, 30, and 40 cycles/min using paired change-score methods and random-effects REML models where applicable. At 20 cycles/min, the pooled mean change in femoral venous blood flow velocity was 15.23 cm/s (95% CI: 10.04 to 18.42; I² = 40.67%). At 30 cycles/min, the pooled mean change was 14.20 cm/s (95% CI: 9.92 to 16.48; I² = 37.90%). At 40 cycles/min, the pooled mean change was 9.50 cm/s (95% CI: 6.93 to 13.06; I² = 32.81%). No conventional overall pooled estimate was calculated across the broader 20–40 cycles/min range to avoid unit-of-analysis error related to repeated frequency comparisons and shared baseline measurements (**Figure 3**).

#### 3.4.4 APE frequency: 60–100 cycles/min

**Figure 4:**
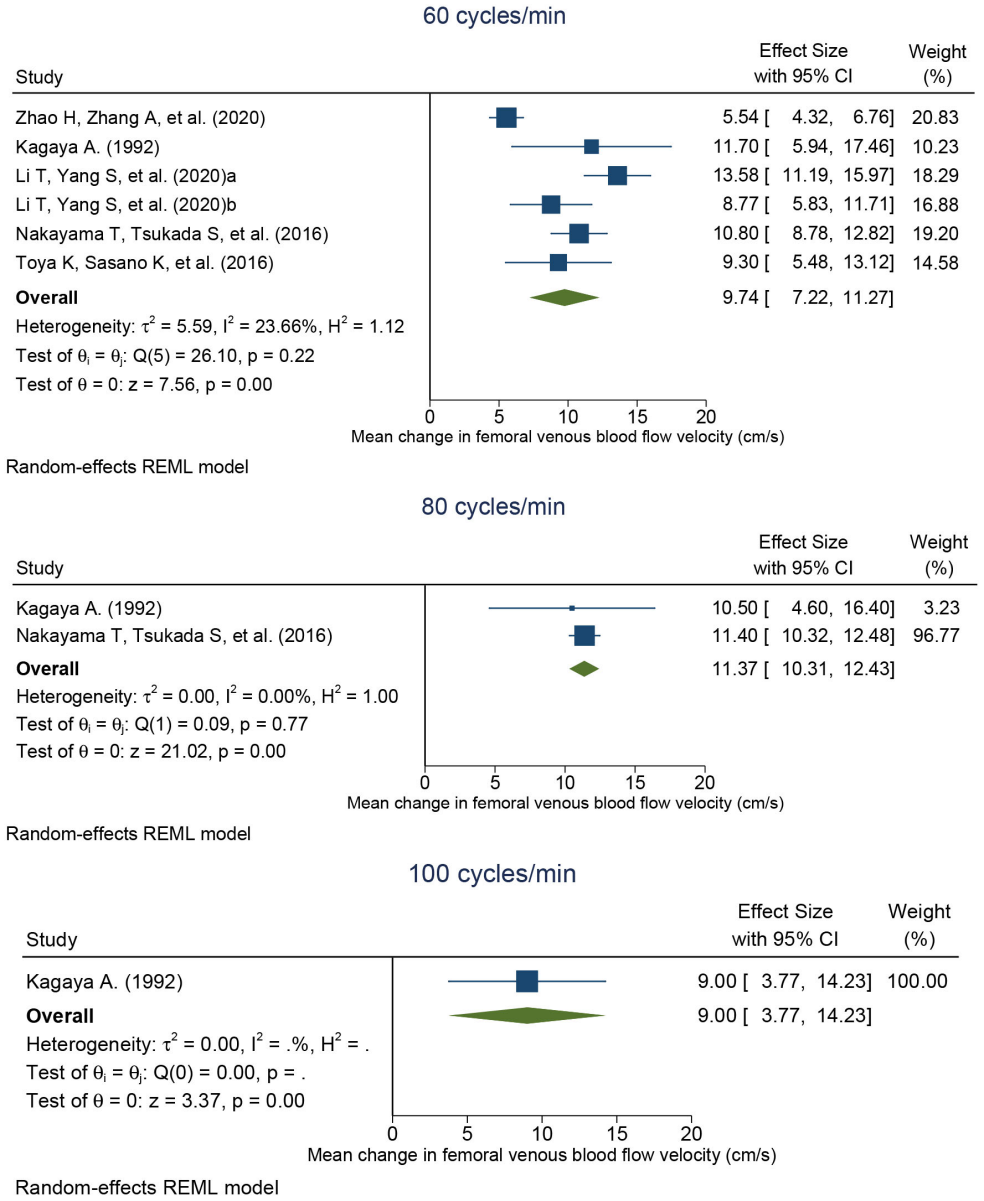
Frequency-specific changes in mean femoral venous blood flow velocity after ankle pump exercise at 60–100 cycles/min. Effect estimates were calculated as paired mean changes from baseline. Frequency-specific estimates are shown for 60, 80, and 100 cycles/min. No conventional overall pooled estimate was calculated across the broader 60–100 cycles/min range to avoid unit-of-analysis error.

In the 60–100 cycles/min frequency range, analyses were conducted at the specific-frequency level for 60, 80, and 100 cycles/min using paired change-score methods and random-effects REML models where applicable. At 60 cycles/min, the pooled mean change in femoral venous blood flow velocity was 9.74 cm/s (95% CI: 7.22 to 11.27; I² = 23.66%). At 80 cycles/min, the pooled mean change was 11.37 cm/s (95% CI: 10.31 to 12.43; I² = 0.00%). At 100 cycles/min, one comparison was available, showing a mean change of 9.00 cm/s (95% CI: 3.77 to 14.23). No conventional overall pooled estimate was calculated across the broader 60–100 cycles/min range to avoid unit-of-analysis error related to repeated frequency comparisons and shared baseline measurements (**Figure 4**).

Overall, these frequency-specific findings were based mainly on within-arm changes from baseline rather than direct head-to-head comparisons between different APE frequencies. Therefore, differences in mean changes across frequencies should be interpreted as descriptive findings rather than formal comparative estimates of frequency effects.

### 3.5 Sensitivity analysis

Sensitivity analyses were performed to assess the robustness of the primary random-effects REML findings. First, fixed-effect inverse-variance models were applied as model-based sensitivity analyses. Consistent with the primary analyses, fixed-effect estimates were calculated within each specific exercise frequency when sufficient independent comparisons were available, and no conventional overall pooled estimates were calculated across the broader frequency ranges. The fixed-effect analyses showed that the direction and statistical significance of the estimates were generally consistent with the primary random-effects analyses across the 3–6, 10–15, 20–40, and 60–100 cycles/min ranges (**Supplementary Figures 1**–**3**).

Second, leave-one-out sensitivity analyses were conducted by sequentially removing each study within the corresponding specific-frequency analyses. Exclusion of any single study did not materially change the direction or statistical significance of the estimates, although small variations in effect size were observed. Overall, the fixed-effect and leave-one-out sensitivity analyses supported the robustness of the main findings.

### 3.6 Certainty of evidence

The certainty of evidence for mean femoral venous blood flow velocity was assessed using the GRADE approach and summarized by predefined frequency range. Overall, the certainty of evidence was rated as very low across all evaluated frequency ranges. The main reasons for downgrading were the predominance of uncontrolled before-and-after studies, limited direct comparisons between different APE frequencies, small numbers of studies or comparisons for several specific frequencies, heterogeneity in participant populations and intervention protocols, and reliance on short-term hemodynamic outcomes rather than clinical endpoints. Therefore, the frequency-specific findings should be interpreted cautiously. The GRADE assessment is presented in **Supplementary Table 4**.

## 4 Discussion

This systematic review and meta-analysis evaluated the effects of APE performed at different frequencies on lower-limb hemodynamics. Using paired change-score methods, the frequency-specific analyses showed that APE was generally associated with increased mean femoral venous blood flow velocity across the evaluated frequencies, suggesting a favorable effect on lower-limb venous hemodynamics. The observed increases were relatively small at lower frequencies, particularly within the 3–6 cycles/min range, whereas larger within-arm increases tended to be observed at frequencies of 10 cycles/min or higher. However, because most included studies provided within-arm before-after data rather than direct comparisons between different exercise frequencies, apparent differences across frequencies may be influenced by between-study differences in participant populations, baseline characteristics, and study methods. Therefore, these findings should not be interpreted as definitive evidence of a dose-response frequency effect. In addition, the available data did not show a consistent pattern indicating that further increases in exercise frequency necessarily produced additional hemodynamic benefits, particularly among the higher-frequency protocols. Because several studies assessed multiple frequencies and some comparisons shared baseline measurements, conventional overall pooled estimates across broader frequency ranges were not calculated to avoid unit-of-analysis error. Sensitivity analyses using fixed-effect models and leave-one-out procedures supported the overall stability of the within-frequency findings. Taken together, these results suggest that APE performed at frequencies of 10 cycles/min or higher may be associated with favorable increases in mean femoral venous blood flow velocity, although the optimal frequency remains uncertain.

The frequency-specific findings showed that APE was associated with increases in mean femoral venous blood flow velocity across the evaluated specific frequencies. The increase was relatively modest at lower frequencies, particularly within the 3–6 cycles/min range, whereas frequencies of 10 cycles/min or higher tended to show larger increases. However, the available evidence did not indicate a consistent dose-response pattern in which progressively higher frequencies produced proportionally greater hemodynamic benefits. This observation is broadly consistent with previous clinical studies suggesting that increasing ankle pump frequency may enhance lower-limb venous return, although the benefit may plateau after a certain frequency threshold is reached (Wnuk et al., 2024). One possible explanation is that higher-frequency APE produces more repeated contractions of the calf muscles within the same period, thereby strengthening the calf muscle pump effect and promoting venous return. Previous studies have shown that a single contraction of normal calf muscle can expel a measurable volume of venous blood, suggesting that faster movement patterns may theoretically increase effective blood displacement over the same duration and improve lower-limb circulation more efficiently (Houghton et al., 2021; Padberg, 2024). At the same time, the relationship between exercise frequency and venous flow improvement is unlikely to be purely linear. Although increasing frequency raises the number of muscle contractions, venous return may approach a plateau once frequency reaches a certain level, making further gains limited (Persichini et al., 2022). This may be related to the rhythm of muscle contraction and relaxation, venous refilling time, movement amplitude, and exercise quality. In clinical practice, the hemodynamic response to APE may depend not only on frequency, but also on whether patients can complete sufficient ankle dorsiflexion and plantarflexion with adequate movement amplitude and regular rhythm. In other words, a frequency that is too low may provide too few effective contractions to optimize venous return, whereas an excessively high frequency may not further improve hemodynamics and could reduce practical benefit because of faster, less complete movements, poorer adherence, or greater muscle fatigue.

Overall, our findings are broadly consistent with previous reports, while also suggesting that the relationship between APE frequency and lower-limb blood flow improvement is unlikely to be simply linear (Toya et al., 2016; Wang et al., 2023). Earlier studies have indicated that faster ankle pump movements may enhance venous return and improve lower-limb hemodynamics, possibly because a single contraction of the normal calf muscle can expel approximately 60–90 mL of blood (Cheung, 2018; Tauraginskii et al., 2023). On this basis, a higher movement frequency may generate more repeated contractions within the same period and thereby promote venous return. However, slower or more traditional protocols are not necessarily inferior in all circumstances. Traditional APE protocols often require the ankle to remain in dorsiflexion and plantarflexion for a certain duration, based on the assumption that longer or more complete contractions may strengthen calf muscle action and enhance the muscle pump effect (Lattimer et al., 2018). It has also been reported that performing one ankle pump movement every 3–4 s may improve venous return while reducing post-exercise fatigue compared with very slow or very fast patterns, making it more feasible and acceptable in clinical practice (Maharem et al., 2022). Other studies have found no significant differences between faster and slower protocols in terms of venous diameter or blood flow velocity. One possible explanation is that although slower movement may increase the strength or completeness of each contraction, prolonged contraction may also keep the vein under sustained compression, thereby partly offsetting its benefit for venous return (O’Riordan, 2022). Taken together, these findings suggest that the hemodynamic effects of APE may be shaped by several interacting factors, including contraction frequency, contraction strength, movement amplitude, venous refilling time, and exercise-related fatigue (Forman, 2020). This may help explain why, in the present analysis, frequencies of 10 cycles/min or higher tended to show more noticeable increases than lower frequencies, whereas progressively higher frequencies did not appear to provide proportional additional benefits.

Although our findings were generally consistent with some previous reports, several limitations should be considered when interpreting the results. The included populations ranged from healthy participants to postoperative patients, individuals with fractures, and patients with hemiplegia, all of whom may differ in calf muscle strength, venous function, mobility status, and baseline hemodynamic characteristics. In addition, the intervention procedures, exercise duration, movement quality, outcome measures, and timing of assessment were not fully consistent across studies. Most included studies used within-arm before-after designs rather than direct randomized or within-participant comparisons between different frequencies, which limits the ability to determine a true dose-response relationship or the optimal APE frequency. Some studies also had small sample sizes and methodological limitations, which may have affected the comparability and stability of the findings. Therefore, the frequency-specific results should be interpreted cautiously. As a simple, low-cost, and easy-to-implement physical intervention, APE may still have practical value for individuals with prolonged bed rest, postoperative immobilization, or restricted mobility. When designing APE protocols, clinicians should consider not only exercise frequency but also movement amplitude, exercise duration, patient tolerance, movement quality, and adherence. Future studies should quantitatively assess tolerance using perceived exertion scales, pain or discomfort scores, fatigue scores, muscle cramp occurrence, adverse events, and the proportion of participants unable to complete the prescribed protocol. Long-term compliance should also be evaluated by recording the percentage of prescribed sessions completed, completed cycles per session, missed sessions, dropout rate, and reasons for discontinuation. Exercise diaries, nurse- or caregiver-supervised logs, wearable sensors, mobile applications, or device-based counters could be used to improve the accuracy of adherence monitoring. These measures would help determine whether a given APE frequency is not only hemodynamically effective but also acceptable and sustainable in routine clinical or home-based practice.

## 5 Limitations

Several limitations should be acknowledged. First, the number of included studies was limited, and only one study was an RCT, whereas most studies used non-randomized, quasi-experimental, or uncontrolled before-and-after designs. This substantially reduces the overall strength of the evidence and limits causal inference. The pooled estimates may be affected by selection bias, confounding, regression to the mean, and differences in measurement conditions. Consistent with this, the GRADE assessment indicated very low certainty of evidence across all evaluated frequency ranges. Second, most included studies reported within-arm pre-post changes rather than direct randomized or within-participant comparisons between different APE frequencies. Therefore, the frequency-specific findings should not be interpreted as definitive evidence of a dose-response relationship or as proof of an optimal exercise frequency. Third, several studies assessed multiple frequencies in the same participants and reused baseline measurements, resulting in non-independent frequency comparisons. Although conventional overall pooling across broader frequency ranges was avoided, the results should still be interpreted cautiously. Finally, differences in baseline characteristics, clinical conditions, calf muscle strength, venous function, mobility status, intervention procedures, movement quality, assessment timing, and outcome measures across studies may have affected the consistency and comparability of the findings and may partly explain differences in velocity changes across frequencies. Further large-scale, well-designed studies directly comparing different APE frequencies are needed to determine the most appropriate exercise protocol.

## 6 Conclusions

In conclusion, APE may improve lower-limb venous hemodynamics by increasing mean femoral venous blood flow velocity. Frequency-specific analyses suggested that APE performed at 10 cycles/min or higher tended to show larger within-arm increases in venous blood flow velocity than lower-frequency protocols. However, because most included studies provided within-arm before-after data rather than direct comparisons between different exercise frequencies, this observation should not be interpreted as definitive evidence of a dose-response frequency effect. The available evidence also did not show a consistent pattern suggesting that further increases in frequency necessarily produce additional hemodynamic benefits. Therefore, frequencies of 10 cycles/min or higher may be considered a potential practical option for achieving favorable circulatory effects in clinical settings. Nevertheless, given the predominance of non-randomized or quasi-experimental evidence and the very low certainty of evidence, these findings should be interpreted cautiously, and the optimal APE protocol remains uncertain. Further high-quality studies are needed to determine the most appropriate exercise protocol, including frequency, duration, movement amplitude, tolerability, adherence, and clinical applicability.

## Supplementary material

Supplementary Figure 1 Fixed-effect sensitivity analyses of frequency-specific changes in mean femoral venous blood flow velocity after ankle pump exercise at 3–6 and 10–15 cycles/min. (A) Frequency-specific estimates at 3, 5, and 6 cycles/min; (B) frequency-specific estimates at 10, 12, and 15 cycles/min. Effect estimates were calculated as paired mean changes from baseline using fixed-effect inverse-variance models and should be interpreted as within-frequency changes rather than formal comparative estimates between frequencies.

Supplementary Figure 2 Fixed-effect sensitivity analyses of frequency-specific changes in mean femoral venous blood flow velocity after ankle pump exercise at 20–40 cycles/min. Frequency-specific estimates are shown for 20, 30, and 40 cycles/min. Effect estimates were calculated as paired mean changes from baseline using fixed-effect inverse-variance models.

Supplementary Figure 3 Fixed-effect sensitivity analyses of frequency-specific changes in mean femoral venous blood flow velocity after ankle pump exercise at 60–100 cycles/min. Frequency-specific estimates are shown for 60, 80, and 100 cycles/min. Effect estimates were calculated as paired mean changes from baseline using fixed-effect inverse-variance models.
