# Supplementary material for: Effects of different frequencies of ankle pump exercise on lower limb hemodynamics: a systematic review and meta-analysis
Source: Front Physiol. 2026 Jul 30;17:1880108. doi: 10.3389/fphys.2026.1880108 (PMC13470180; doi:10.3389/fphys.2026.1880108)
Supplement: Supplementary file 1 [file Table1.docx]

**Supplementary Table 1.** Search Strategy.

#1 lower extremity hemodynamics [Title/Abstract]

#2 blood flow rate [Title/Abstract]

#3 blood volume [Title/Abstract]

#4 venous blood return [Title/Abstract]

#5 #1 OR #2 OR #3 OR #4

#6 ankle pump exercise [Title/Abstract]

#7 functional ∗training [Title/Abstract]

#8 functional ∗exercise [Title/Abstract]

#9 ankle motion [Title/Abstract]

#10 ankle exercise [Title/Abstract]

#11 ankle plantarflexion [Title/Abstract]

#12 ankle valgus [Title/Abstract]

#13 #6 OR #7 OR #8 OR #9 OR #10 OR #11 OR #12

#14 (randomized controlled trial[pt] OR controlled clinical trial[pt] OR randomized[tiab] OR placebo[tiab] OR clinical trials as topic[mesh:noexp] OR randomly[tiab] OR trial[ti]) NOT (animals [mh] NOT (humans [mh] AND animals[mh]))

#15 #5 And #13 And #14
