# Supplementary material for: Effects of different frequencies of ankle pump exercise on lower limb hemodynamics: a systematic review and meta-analysis
Source: Front Physiol. 2026 Jul 30;17:1880108. doi: 10.3389/fphys.2026.1880108 (PMC13470180; doi:10.3389/fphys.2026.1880108)
Supplement: Supplementary file 2 [file Table3.docx]

**Supplementary Table 3.** Mapping of included studies, exercise frequencies, and frequency-specific comparisons used in the meta-analysis.

| Study | Frequency range | Specific frequency | Participant group/arm | N | Treated as an independent comparison? | Included in specific-  frequency analysis |
| --- | --- | --- | --- | --- | --- | --- |
| Han J, Wang Y, et al. (2024)a | 3–6 cycles/min | 3 | Independent participant group a | 30 | Yes, independent participant group | Yes |
| Han J, Wang Y, et al. (2024)b | 3–6 cycles/min | 3 | Independent participant group b | 30 | Yes, independent participant group | Yes |
| Li H, Zhang W, et al. (2022) | 3–6 cycles/min | 3 | Independent frequency arm | 153 | Yes, independent frequency arm | Yes |
| Zhou S, Ma Q, et al. (2020) | 3–6 cycles/min | 5 | Frequency-specific comparison | 11 | Yes, single comparison | Yes |
| Han J, Wang Y, et al. (2024)a | 3–6 cycles/min | 6 | Independent participant group a | 30 | Yes, independent participant group | Yes |
| Han J, Wang Y, et al. (2024)b | 3–6 cycles/min | 6 | Independent participant group b | 30 | Yes, independent participant group | Yes |
| Zhao H, Zhang A, et al. (2020) | 3–6 cycles/min | 6 | Independent frequency arm | 63 | No, not independent across frequencies | Yes |
| Li T, Yang S, et al. (2020)a | 3–6 cycles/min | 6 | Independent participant group a | 30 | Yes, independent participant group | Yes |
| Li T, Yang S, et al. (2020)b | 3–6 cycles/min | 6 | Independent participant group b | 30 | Yes, independent participant group | Yes |
| Zhou S, Ma Q, et al. (2020) | 10–15 cycles/min | 10 | Independent frequency arm | 11 | Yes, independent frequency arm | Yes |
| Li T, Yang S, et al. (2020)a | 10–15 cycles/min | 10 | Independent participant group a | 30 | Yes, independent participant group | Yes |
| Li T, Yang S, et al. (2020)b | 10–15 cycles/min | 10 | Independent participant group b | 30 | Yes, independent participant group | Yes |
| Toya K, Sasano K, et al. (2016) | 10–15 cycles/min | 10 | Independent frequency arm | 10 | Yes, independent frequency arm | Yes |
| Zhao H, Zhang A, et al. (2020) | 10–15 cycles/min | 12 | Independent frequency arm | 63 | Yes, independent frequency arm | Yes |
| Han J, Wang Y, et al. (2024)a | 10–15 cycles/min | 15 | Independent participant group a | 30 | Yes, independent participant group | Yes |
| Han J, Wang Y, et al. (2024)b | 10–15 cycles/min | 15 | Independent participant group b | 30 | Yes, independent participant group | Yes |
| Zhao H, Zhang A, et al. (2020) | 10–15 cycles/min | 15 | Independent frequency arm | 63 | Yes, independent frequency arm | Yes |
| Zhou S, Ma Q, et al. (2020) | 10–15 cycles/min | 15 | Independent frequency arm | 11 | Yes, independent frequency arm | Yes |
| Zhao H, Zhang A, et al. (2020) | 20–40 cycles/min | 20 | Independent frequency arm | 63 | Yes, independent frequency arm | Yes |
| Kagaya A. (1992) | 20–40 cycles/min | 20 | Independent frequency arm | 6 | Yes, independent frequency arm | Yes |
| Han J, Wang Y, et al. (2024)a | 20–40 cycles/min | 30 | Independent participant group a | 30 | Yes, independent participant group | Yes |
| Han J, Wang Y, et al. (2024)b | 20–40 cycles/min | 30 | Independent participant group b | 30 | Yes, independent participant group | Yes |
| Zhao H, Zhang A, et al. (2020) | 20–40 cycles/min | 30 | Independent frequency arm | 63 | Yes, independent frequency arm | Yes |
| Li H, Zhang W, et al. (2022) | 20–40 cycles/min | 30 | Independent frequency arm | 154 | Yes, independent frequency arm | Yes |
| Li T, Yang S, et al. (2020)a | 20–40 cycles/min | 30 | Independent participant group a | 30 | Yes, independent participant group | Yes |
| Li T, Yang S, et al. (2020)b | 20–40 cycles/min | 30 | Independent participant group b | 30 | Yes, independent participant group | Yes |
| Toya K, Sasano K, et al. (2016) | 20–40 cycles/min | 30 | Frequency-specific comparison | 10 | Yes, single comparison | Yes |
| Kagaya A. (1992) | 20–40 cycles/min | 40 | Frequency-specific comparison | 6 | Yes, single comparison | Yes |
| Nakayama T, Tsukada S, et al. (2016) | 20–40 cycles/min | 40 | Frequency-specific comparison | 29 | Yes, single comparison | Yes |
| Zhao H, Zhang A, et al. (2020) | 60–100 cycles/min | 60 | Frequency-specific comparison | 63 | Yes, single comparison | Yes |
| Kagaya A. (1992) | 60–100 cycles/min | 60 | Frequency-specific comparison | 6 | Yes, single comparison | Yes |
| Li T, Yang S, et al. (2020)a | 60–100 cycles/min | 60 | Independent participant group a | 30 | Yes, independent participant group | Yes |
| Li T, Yang S, et al. (2020)b | 60–100 cycles/min | 60 | Independent participant group b | 30 | Yes, independent participant group | Yes |
| Nakayama T, Tsukada S, et al. (2016) | 60–100 cycles/min | 60 | Frequency-specific comparison | 29 | Yes, single comparison | Yes |
| Toya K, Sasano K, et al. (2016) | 60–100 cycles/min | 60 | Frequency-specific comparison | 10 | Yes, single comparison | Yes |
| Kagaya A. (1992) | 60–100 cycles/min | 80 | Frequency-specific comparison | 6 | Yes, single comparison | Yes |
| Nakayama T, Tsukada S, et al. (2016) | 60–100 cycles/min | 80 | Frequency-specific comparison | 29 | Yes, single comparison | Yes |
| Kagaya A. (1992) | 60–100 cycles/min | 100 | Frequency-specific comparison | 6 | Yes, single comparison | Yes |

Note: The labels “a” and “b” for Han J, Wang Y, et al. (2024) and Li T, Yang S, et al. (2020) indicate independent participant groups. Therefore, these comparisons were treated as independent within the same specific-frequency analysis. Comparisons derived from repeated measurements of different exercise frequencies within the same participants were not treated as independent across frequencies and were not pooled into conventional overall estimates across broader frequency ranges. The total sample size of 546 refers to unique participants across the included studies rather than the sum of all frequency-specific comparisons.
