# Supplementary material for: Effects of different frequencies of ankle pump exercise on lower limb hemodynamics: a systematic review and meta-analysis
Source: Front Physiol. 2026 Jul 30;17:1880108. doi: 10.3389/fphys.2026.1880108 (PMC13470180; doi:10.3389/fphys.2026.1880108)
Supplement: Supplementary file 3 [file Table4.docx]

**Supplementary Table 4.** GRADE assessment of the certainty of evidence for mean femoral venous blood flow velocity by predefined frequency range.

| Frequency range | Specific frequencies included | Outcome | No. of studies/comparisons | Effect direction | Certainty of evidence | Reason for downgrading |
| --- | --- | --- | --- | --- | --- | --- |
| 3–6 cycles/min | 3, 5, and 6 cycles/min | Mean femoral venous blood flow velocity | [6 studies / 9 comparisons] | Increased from baseline across  specific-frequency analyses | ⊕⊝⊝⊝ Very low | Downgraded for serious study limitations because most evidence came from uncontrolled before-after studies; serious indirectness because evidence reflected short-term hemodynamic changes rather than clinical outcomes and did not provide direct frequency comparisons; and serious imprecision because several specific frequencies were supported by few comparisons. |
| 10–15 cycles/min | 10, 12, and 15 cycles/min | Mean femoral venous blood flow velocity | [7 studies / 9 comparisons] | Increased from baseline across specific-frequency analyses | ⊕⊝⊝⊝ Very low | Downgraded for serious study limitations, limited direct comparisons between frequencies, heterogeneous populations and protocols, and imprecision due to small numbers of comparisons at some specific frequencies. |
| 20–40 cycles/min | 20, 30, and 40 cycles/min | Mean femoral venous blood flow velocity | [8 studies / 11 comparisons] | Increased from baseline across specific-frequency analyses | ⊕⊝⊝⊝ Very low | Downgraded for serious study limitations, repeated-frequency measurements with shared baselines in some studies, heterogeneity in populations and intervention procedures, and limited direct comparative evidence. |
| 60–100 cycles/min | 60, 80, and 100 cycles/min | Mean femoral venous blood flow velocity | [8 studies / 9 comparisons] | Increased from baseline across specific-frequency analyses | ⊕⊝⊝⊝ Very low | Downgraded for serious study limitations, repeated-frequency measurements with shared baselines, small numbers of comparisons at some frequencies, and reliance on short-term hemodynamic outcomes. |

Note: Certainty of evidence was summarized by predefined frequency range for presentation. Meta-analytic estimates were reported at the specific-frequency level, and no conventional overall pooled estimates were calculated across broader frequency ranges. The certainty ratings reflect confidence in the evidence within each predefined frequency range rather than direct comparative certainty between frequencies.
